# Marked underutilization of lipoprotein(a) testing in peripheral artery disease: a national analysis of 8.3 million patients

**DOI:** 10.1016/j.ajpc.2026.101628

**Published:** 2026-04-09

**Authors:** Mustafa Naguib, Ava J. Fascetti, Elsie G. Ross, Edward J. Wang, Michael Wilkinson, Ehtisham Mahmud, Pam Taub, Harpreet S. Bhatia, Mattheus Ramsis

**Affiliations:** a School of Medicine, University of California San Diego, La Jolla, CA, USA; b The Design Lab, Department of Electrical and Computer Engineering, University of California San Diego, La Jolla, CA, USA; c Division of Vascular Surgery, Department of Surgery, University of California San Diego, La Jolla, CA, USA; d Division of Cardiovascular Medicine, Department of Medicine, University of California San Diego, La Jolla, CA, USA

## 1 Background

Lipoprotein(a) [Lp(a)] is an atherogenic, genetically determined risk factor for cardiovascular diseases including peripheral artery disease (PAD), coronary artery disease (CAD), and ischemic stroke [1]. About 1 in 5 individuals worldwide and 1 in 4 individuals with atherosclerotic cardiovascular disease (ASCVD) have an elevated Lp(a) >50 mg/dL (>125 nmol/L), which is associated with worsening PAD and increased likelihood of major adverse limb events (MALE) [2,3]. Data on national testing trends of Lp(a) demonstrate increasing but low testing in the general population and in young ischemic stroke patients, and guideline recommendations for testing vary between organizations [[4], [5], [6]]. Despite the relationship between Lp(a) and PAD, little data on national testing trends of Lp(a) in patients with PAD are available. Information on national testing trends of Lp(a) in this population may influence future guideline implementation recommendations to increase Lp(a) testing. This study aims to use a large, real-world dataset to assess trends of Lp(a) testing in PAD patients in the United States from 2015–2025.

## 2 Methods

We performed a retrospective analysis of lipoprotein(a) testing in patients with peripheral artery disease across the United States from January 1, 2015 to December 31, 2025 using Epic Cosmos, a nationwide, de-identified electronic health record (EHR) dataset. PAD was defined by the presence of ≥1 ICD-10-CM diagnosis code consistent with peripheral artery disease recorded in encounter diagnoses and/or the problem list within Epic Cosmos, reflecting world clinical documentation across a large, multi-institutional EHR network and has been widely used in population-level studies [7]. Lp(a) testing was defined by the presence of a recorded laboratory result within the dataset.

Testing rates were calculated as the number of distinct patients undergoing Lp(a) testing per year divided by the number of PAD patients with at least one healthcare encounter within that year. We also evaluated overall testing prevalence, geographic variation, and testing rates stratified by age, sex, race, ethnicity, social vulnerability index (SVI), and presence of CAD. For each stratum, we calculated the proportion tested with Wilson 95% confidence intervals and assessed between group differences using chi-square or Fisher exact tests as appropriate. Geographic variation was visualized using a state-level heatmap representing the proportion of total Lp(a) tests contributed by each state. All analyses were descriptive and intended to characterize population-level patterns of PAD within the Cosmos network rather than infer causal associations. All data are de-identified in compliance with HIPAA standards and governed under Epic’s “Rules of the Road” for institutional data use.

## 3 Results

**Fig. 1:**
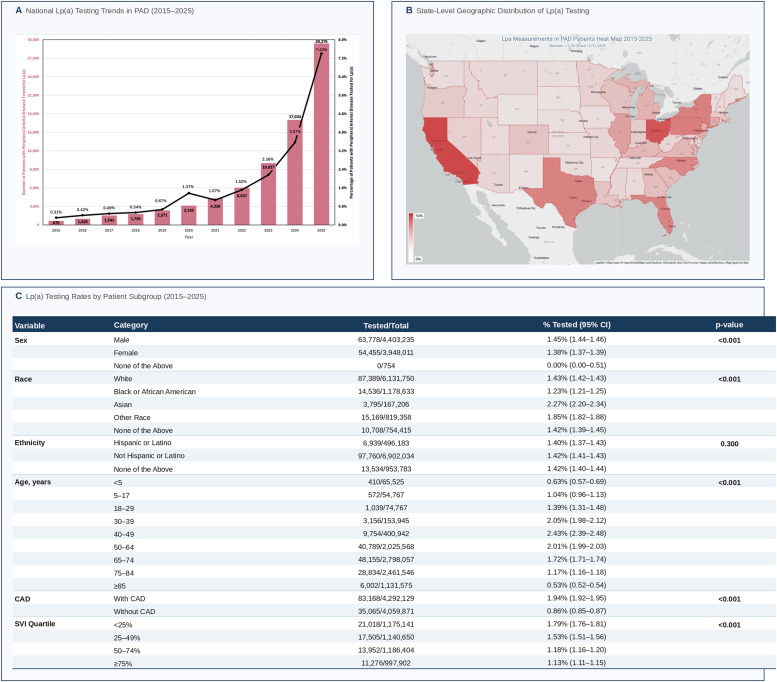
**National Trends, Geographic Distribution, and Subgroup Variation in Lipoprotein(a) Testing Among U.S. Patients with Peripheral Artery Disease (2015-2025). Panel A:** Yearly trends in distinct Lp(a) testing in patients with peripheral artery disease in the United States, showing a substantial increase from 2015 to 2025. **Panel B:** Geographic variation was visualized using a state-level heatmap representing the proportion of total Lp(a) tests contributed by each state and created using Epic Cosmos. Analysis performed by Mattheus Ramsis, MD, and internally reviewed by Mattheus Ramsis, MD at UCSD Health on 02/06/26. **Panel C:** Lp(a) testing proportions (95% Cls) stratified by sex, race, ethnicity, age, CAD status, and Social Vulnerability Index (SVI) quartile; p-values from chi-square tests for overall group differences.

From 2015 to 2025, 118,233 of a total of 8352,000 distinct patients with peripheral artery disease (1.42%) underwent Lp(a) testing. Additionally, the annual number of tested patients increased significantly from 679 in 2015 to 29,375 in 2025 (*p* < 0.001), and the annual percentage of patients undergoing Lp(a) testing increased from 0.31 % in 2015 to 7.40 % in 2025, as shown in Fig. 1. The states with the largest number of tested patients were California (9.91 %), Ohio (9.13 %), and Pennsylvania (6.44 %). Among tested patients, those aged 65–75 years comprised the largest percentage of tested patients (48,155; 34.7 %); however, the highest rate of testing occurred in patients aged 40–50 (9754; 2.43 %). Between sexes, significantly more men with PAD (63,778; 1.45 %) were tested compared to women (54,455; 1.38 %). Analyzing patients with reported racial data, patients who identified as Asian underwent testing for Lp(a) at the highest rate (2.27 %), followed by patients who identified as Other Race (1.85 %), White (1.43 %), “None of the above” (1.42 %), and Black or African American (1.23 %). Among reported ethnic identities, rates of testing were similar among patients who identified as Hispanic or Latino (1.40 %) and those who identified as non-Hispanic (1.42 %). PAD patients who had a concurrent diagnosis of CAD underwent Lp(a) testing at a higher rate (1.94 %) than those who did not have CAD (0.86 %). Furthermore, of those tested who had a reported SVI, patients from communities with an SVI <25 % had the highest testing rates (1.79 %), decreasing by quartile with the lowest testing rates among communities with an SVI ≥75 % (1.13 %).

## 4 Discussion

In this large national analysis of >8 million patients with peripheral artery disease, we observed substantial growth in Lp(a) testing over the past decade. Despite this increase, overall testing remains extremely limited, with only 1.42 % (less than one in 70) PAD patients nationally having ever undergone measurement. These findings highlight a substantial implementation gap between emerging guideline recommendations and real-world clinical practice [5].

Demographic analyses show statistically significant differences in test performance across groups stratified by sex, race, and SVI. In particular, lower rates of testing were identified in female patients, Black patients, and patients with a higher SVI. Discrepancies in testing in these groups may be related to differences in healthcare access, including availability of resources, specialists, and systems that routinely screen patients with PAD for elevated Lp(a). These patterns may also reflect variation in provider awareness or practice patterns, as well as broader system-level factors that influence the implementation of guideline-recommended testing. Additionally, these discrepancies, as well as differences in geographic distribution, may be partially attributed to which institutions and clinics have their data included in Epic Cosmos.

Additionally, the age group with the highest rate of testing was those aged 40–50. Younger patients with PAD may be undergoing testing for elevated Lp(a) at a higher rate due to several factors, such as more extensive testing of risk and higher concerns for genetic etiologies of dyslipidemia. Furthermore, significantly more patients with both PAD and CAD were tested for Lp(a) than those with PAD alone. Factors contributing to this difference may potentially involve increased awareness of guidelines emphasizing Lp(a) testing in CAD patients, a larger body of evidence linking Lp(a) to coronary events than peripheral events, and inclusion of specialists and lipid clinics more frequently testing CAD patients as a primary or secondary screening tool.

As Lp(a)-targeted therapies continue to advance in clinical development, improved identification of patients with elevated Lp(a) may become increasingly important for preventive cardiovascular care. Standardized screening approaches, increased provider awareness, and the integration of electronic health record–based decision support tools may improve the implementation of Lp(a) testing and reduce differences in access to risk assessment.

Notably, PAD was defined using diagnosis-based coding within the Epic Cosmos dataset, which may introduce variability in diagnostic certainty and coding practices across sites. More restrictive definitions may improve specificity but are not uniformly available and may reduce generalizability.

Additionally, Lp(a) testing performed outside participating systems or not interfaced into the EHR may not be captured, and therefore, testing rates may be underestimated. Accordingly, these findings are best interpreted as reflecting population-level patterns of documented testing rather than complete capture of all testing events.

## 5 Conclusion

In this national analysis of >8 million patients with peripheral artery disease, Lp(a) testing increased substantially over the past decade but remains markedly underutilized. Significant differences in testing persist across demographic and socioeconomic groups, highlighting ongoing gaps between guideline recommendations and real-world clinical implementation. Leveraging EHR-based strategies, including clinical decision support tools and population health registries, may facilitate more systematic Lp(a) screening, improve adherence to guideline-recommended testing, and help reduce differences in preventive cardiovascular care.

## Disclosure statement

Dr. Pam Taub is supported by NIH grant 2R01DK118278–06A1 and serves as a consultant to Amgen, Boehringer Ingelheim, Lilly, Novo Nordisk, Novartis, Medtronic, Jazz, Bayer, Arrowhead, and Roche.

Dr. Harpreet Bhatia is supported by NIH grant 1K08HL166962 and serves as a consultant/advisor for Kaneka, Novartis, NewAmsterdam, Arrowhead, and Abbott.

Dr. Michael Wilkinson is supported by the BEACON Lp(a) Research Center at UC San Diego and by the National Institutes of Health grants 1R01DK118278–01 and R01DK139356 and by a grant from the Larry L. Hillblom Foundation. He is a consultant to Amarin, Regeneron, Kaneka, Ionis, and NewAmsterdam; reports advisory board fees from Novartis, Regeneron, Ionis, Arrowhead, and NewAmsterdam; received grant support from Amgen and Esperion (investigator-initiated studies) and support through an institutional consulting agreement with Novartis paid to his institution for advising on research; and has contracted research grants to his institution with Amgen, Novartis, Ionis, Lilly, Silence, and Mineralys.

All other authors have no relevant disclosures.

## Funding statement

This research was supported by the UC San Diego BEACON. No industry sponsorship was involved in the conduct or interpretation of this study.

## Author agreement statement

We the undersigned declare that this manuscript is original, has not been published before and is not currently being considered for publication elsewhere.

We confirm that the manuscript has been read and approved by all named authors and that there are no other persons who satisfied the criteria for authorship but are not listed. We further confirm that the order of authors listed in the manuscript has been approved by all of us.

We understand that the Corresponding Author is the sole contact for the Editorial process. He is responsible for communicating with the other authors about progress, submissions of revisions and final approval of proofs.

Mustafa Naguib, MTM

Email: mhnaguib@health.ucsd.edu

Ava J. Fascetti, MS

Email: afascetti@ucsd.edu

Elsie G. Ross, MD

Email: e5ross@health.ucsd.edu

Edward J. Wang, PhD

Email: ejaywang@ucsd.edu

Michael Wilkinson, MD

Email: mjwilkinson@health.ucsd.edu

Ehtisham Mahmud, MD

Email: emahmud@health.ucsd.edu

Pam Taub, MD

Email: ptaub@health.ucsd.edu

Harpreet S. Bhatia, MD

Email: hsbhatia@health.ucsd.edu

Mattheus Ramsis, MD

Email: mramsis@ucsd.edu

## CRediT authorship contribution statement

**Mustafa Naguib:** Writing – review & editing, Writing – original draft, Visualization, Validation, Project administration, Methodology, Investigation, Formal analysis, Conceptualization. **Ava J. Fascetti:** Writing – review & editing, Project administration, Methodology, Investigation. **Elsie G. Ross:** Writing – review & editing, Project administration, Methodology, Investigation. **Edward J. Wang:** Writing – review & editing, Project administration, Methodology, Investigation. **Michael Wilkinson:** Writing – review & editing, Project administration, Methodology, Investigation. **Ehtisham Mahmud:** Writing – review & editing, Project administration, Methodology, Investigation. **Pam Taub:** Writing – review & editing, Project administration, Methodology, Investigation. **Harpreet S. Bhatia:** Writing – review & editing, Project administration, Methodology, Investigation. **Mattheus Ramsis:** Writing – review & editing, Validation, Supervision, Software, Resources, Project administration, Methodology, Investigation, Formal analysis, Data curation, Conceptualization.

## Declaration of competing interest

The authors declare the following financial interests/personal relationships which may be considered as potential competing interests: Harpreet S. Bhatia, MD, is an editor for the American Journal of Preventive Cardiology. In accordance with journal policy, he had no involvement in the peer review or editorial decision-making for this manuscript. The editorial process for this submission was handled independently by another editor.
